# Editorial: Sports and Active Living During the Covid-19 Pandemic

**DOI:** 10.3389/fspor.2021.714986

**Published:** 2021-07-02

**Authors:** Solfrid Bratland-Sanda, Richard Giulianotti, Eva Maria Støa, Tommy Langseth, Simon Rosenbaum

**Affiliations:** ^1^Department of Sport, Physical Education and Outdoor Science, University of South-Eastern Norway, Bø, Norway; ^2^School of Sport, Exercise & Health Sciences, Loughborough University, Loughborough, United Kingdom; ^3^Faculty of Medicine and Health, School of Psychiatry, UNSW Sydney, Kensington, NSW, Australia

**Keywords:** COVID-19, health, athlete, exercise, development

In early January 2020 some concerning news reached the world regarding a newly detected coronavirus (soon classified as Covid-19) in Wuhan, China, which led to serious illness and great risk of death (Taylor, 2021). The highly infectious virus spread rapidly and on 11 March 2020, the World Health Organization (WHO) declared a pandemic. By the end of that month, a large part of the world had imposed “lockdowns” with diverse restrictions on social life to control the spread of the virus and to protect national medical systems (World Health Organization, 2020). By the end of June 2020, Covid-19 had caused at least 500,000 deaths worldwide, and brought nations to a halt, in work, education, travel, recreation, and sport (Fund, 2020).

In this Research Topic, we focus on “Sports and Active Living During the Covid-19 Pandemic,” with particular reference to the unprecedented global shutdown. As editors, in our call for papers, and in planning and developing this Research Topic at the height of the pandemic in 2020, we appreciated the impacts of Covid-19 on sport and active living were complex, profound, and highly uneven. Sport mega-events, leagues, tournaments, and fixtures were postponed or canceled. The biggest casualties were the Tokyo 2020 Olympic Games, the 2020 European Championships in men's football, numerous world championships or “major” events in many other sports (such as golf, tennis, and Formula 1 motor racing), and the world's most popular sport leagues. Gyms, fitness centers, rehabilitation centers, and sport clubs were closed, in many cases permanently due to the loss of participants and income. The closure of schools and educational centers required physical education classes to be suspended or delivered in alternative ways, including online. Sport-related businesses faced bankruptcy, or “went under,” with employees in the sport, exercise and leisure sectors losing their jobs. At the same time, while online fitness and exercise training boomed and are now listed as the top trend of fitness in 2021 (Thompson, 2021), the loss of access to fitness facilities or exercise spaces has had huge health and well-being consequences for individuals and diverse populations.

Although Covid-19 has had a global impact, with many nations following the same general path in terms of lockdowns and social distancing, it has affected different population groups, nations, and regions in different ways with different intensities. The disproportionate impact on marginalized and vulnerable populations made Richard Horton describe the situation as a *syndemic* as opposed to a pandemic (Horton, 2020). This has particularly impacted the global South, with progress on eradicating extreme poverty and development being pushed back decades (Yadav et al., 2020; Rasul et al., 2021). In the global North, people who are older, with underlying health conditions, socio-economically disadvantaged, or from ethnic minority backgrounds, have been most adversely affected by Covid-19, and have had fewer opportunities to engage in outdoor physical activities. Some nations have had several weeks of social distancing, and even curfew, with substantial impacts on lifestyle behaviors, health and well-being, and negative effects on clubs and organizations in sport and outdoor life. For individuals with severe illnesses due to Covid-19 infections, there will be long-term uncertainties about the potential role of exercise with respect to rehabilitation.

The global impact of this pandemic is mirrored in the scale and the transnational reach of our Research Topic, on Sports and Active Living during the Covid-19 Pandemic. The Research Topic has attracted 33 individual articles, which reflects the global urgency and critical importance of this area of academic inquiry. Four of the Frontiers journals participated in this Research Topic: *Frontiers in Sports and Active Living, Frontiers in Psychology, Frontiers in Physiology*, and *Frontiers in Nutrition*. This registers the multidisciplinary significance of the Research Topic. A total of 139 authors from 19 countries representing Africa, Asia, Europe, Oceania, North America, and South America contributed. The resulting papers address both general and specific sports (football/soccer, cycling, golf, rugby, boxing, and tennis), occupational activity, leisure-time activity, and rehabilitation. The population groups which are covered include athletes, exercise professionals such as personal trainers, children and adolescents in homeschooling, students, the older adults, clinical populations, and people with disabilities. The editorial team of this Research Topic seeks to acknowledge that most of these papers were written and submitted during the first phase of the pandemic (May to July 2020), at a time when several of us were balancing our academic work with other requirements and responsibilities such as homeschooling, working from home, and isolating and social distancing in line with different national regulations in force during that period.

## The Research Topic Articles: Five Main Areas of Research

The 33 papers in this Research Topic cover a vast range of sports, areas of active living, themes and issues, and nations and regions. Notwithstanding this diversity, here we identify five broad (and often overlapping) areas of research within these articles with respect to the impacts of Covid-19 on sport and active living.

First, a major area of interest for several papers relates to the impacts of Covid-19, and in particular the consequences of lockdowns on physical fitness, activity, and exercise regimes (Kaur et al.; López-Valenciano et al.; Kim et al.; Pedersen et al.; Zinner et al.; Roberts et al.; Girardi et al.). For example, Kaur et al. found in their study of “fitness freaks” that after initial periods of “negative situational perception,” the participants found motivation to exercise at home. Kim et al. identified constraints—such as time, money, and “work-leisure conflicts” —that could adversely affect participation of some people in physical activities during the pandemic. Research also examined the Covid-19 consequences for elite-level or well-trained sport participants (Pedersen et al.; Zinner et al.; Roberts et al.; Seshadri et al.; Fox-Harding et al.). Zinner et al. also disclosed how for elite canoeists and kayakers, the Covid-19 lockdown led to less training, shorter training sessions, more strength-based training, and more sleep and rest.

Looking beyond athletes and participants, the Covid-19 lockdowns had profound impacts for those on the supply side of the sport, physical activity, and fitness industries. Wright et al. examined the consequences of Covid-19 lockdowns for one “active lifestyle entrepreneur” who was forced to re-evaluate his deep personal and occupational commitments to this sector. Bratland-Sanda et al. studied how Covid-19 restrictions had affected personal trainers in Norway. They found that the lockdowns had generally led to drops in client numbers, income, working and living conditions, and vitality, with women more adversely impacted.

A second area of research inquiry for several papers centered on organizational responses to Covid-19, and their impacts on training and fitness (Roberts et al.; Washif et al.). For example, Washif et al. examined the effects of “quarantine” training camp with world-class athletes. Their findings pointed to a host of benefits, notably in regard to training, nutrition, mental and physical health, and sleep behavior, that were afforded by quarantine camps compared to the players' lockdown experiences. Seshadri et al. examined the physical effects of post-lockdown returns to elite-level competitive sport, with reference to injury rates in men's football's German Bundesliga. They found that returning players were more than three times as likely to pick up injuries, especially muscle injuries, compared to before the Covid lockdown. Finally, Mota et al. draw our attention to how sport governing bodies need to adjust some of the laws and structures within sports in response to the impacts of Covid-19. Specifically, they highlighted the need for more substitutions in football (soccer) to be permitted, particularly due to the more congested fixture schedules and the likely risk of injuries after the lifting of restrictions on sport.

Third, a large volume of research was focused on the impact of Covid-19 on sport participation and physical activities of specific population groups, particularly those that are relatively marginalized or vulnerable in different societies (Kamyuka et al.; Roe et al.; Frahsa et al.; Lozano et al.; Bruinvels et al.; Grant et al.). Kamyuka et al. highlighted how social restrictions due to Covid-19 had adversely affected the physical activities of people with disabilities, notably by increasing perceived isolation and mental ill health. In their study of schoolchildren, Roe et al. found that home-schooling during Covid-19 had distinct impacts on physical activity. Students who were more active were older and tended to be more involved in schoolwork more generally, while parents indicated their unease with greater levels of sedentariness among their children. In regard to studies of older adults (Frahsa et al.; Grant et al.), Frahsa et al., for example, examined the promotion of physical activity in nursing homes during the pandemic and found that this service needed to be more fully embedded within the organizational structures of these settings. Moreover, Covid-19 has highlighted significant gender divisions and inequalities in sport contexts (Lozano et al.; Bruinvels et al.). Lozano et al. examined how in the context of football in Argentina, Covid-19 had exacerbated inequalities; they recommended a range of ways in which these divisions should be addressed, particularly with regard to better resources and recognition. Dixon et al. turn our attention to the “sport for development” (SfD) sector, which uses sport as a development tool primarily in developing and marginalized communities. In examining an SfD agency in Kenya, they found that, for participants, the impending Covid-19 pandemic was bringing some clear problems and challenges (such as restricted activities or home difficulties), but also had some positive aspects (such as family time or rest). Finally, in their opinion piece on youth sport, Kelly et al. contend that Covid-19 represents a “watershed moment” which raises a host of considerations, along methodological, contextual, and practical lines, that need to be addressed by scholars and practitioners in this field. We would argue that such insights apply not only to youth sport, but to all domains of sport and active living. For this area of research, Sorbie et al. provided a data report on golf-related engagement in 1,273 golfers during quarantine restrictions in May 2020. In accordance with the ideal of open science, these data are made available for other researchers to explore.

A fourth realm of research related to the media and communication aspects of sports and active living during the pandemic. Some contributors examined the role of online training tools among different types of athletes, such as cyclists and boxers (Moreno-Tenas et al.; Tjønndal). For example, in their investigation of cyclists in Spain, Moreno-Tenas et al. found that the use of online training tools during Covid-19 confinements served to increase the training frequency of these sport participants. Giulianotti and Collison used a structuralist theoretical approach to identify the key themes and narratives in how the UK mass media reported on sport with respect to Covid-19, often in tried-and-tested ways, for example by claiming to act in the public interest while running regular exposé stories on sport celebrities. Godefroy explored the role of social media in shaping public physical activity during the pandemic, specifically with respect to Instagram influencers.

A fifth area of research related to how Covid-19 impacted on the mood, coping, and well-being of sport participants (Turner et al.; Ronkainen et al.; Sorbie et al.; Xiang et al.; Wright et al.). Positively, Turner et al. disclosed that among middle-aged and senior tennis players in Australia, there was a decrease in the time that was spent on playing and training, but the “emotional well-being” of the participants was not adversely impacted. Xiang et al. and found Wright et al. that different types of physical activity had beneficial impacts on the mental health and well-being of young people during the pandemic.

## Concluding Comments

As we write, we are now in May 2021, and the WHO has registered more than 150 million infections and more than 3 million deaths due to Covid-19 (World Health Organization, 2021). Thanks to the massive efforts of researchers from all over the world, we know more about how we can prevent infections, about the psychosocial consequences of lockdowns and social distancing, and most importantly, about the different efficacies of diverse vaccines. More than 1 billion vaccine doses have been administered globally, and this vaccination rate provides optimism with regard to ending the pandemic. At the same time, Covid-19 has forced us to think about the risks of future pandemics or other global shocks, and how we may be better positioned to withstand their impacts in all areas of social life.

This Research Topic has provided us with a wide range and depth of knowledge on how the initial part of the Covid-19 pandemic impacted upon many areas of sport and active living. Further research in this area is essential. Follow-up studies will advance our knowledge of the medium- and longer-term impacts of Covid-19 on sports and active living. Moreover, the diverse research provided by this Research Topic, and by further scholarship, will leave us in a stronger position to understand, and to withstand, the impacts of these future, inevitable global challenges.

## Author Contributions

SB-S and RG drafted the manuscript. TL, ES, and SR commented and revised the draft. All authors approved the final version of the manuscript.

## Conflict of Interest

The authors declare that the research was conducted in the absence of any commercial or financial relationships that could be construed as a potential conflict of interest.
